# Metformin reduces morphine tolerance by inhibiting microglial-mediated neuroinflammation

**DOI:** 10.1186/s12974-016-0754-9

**Published:** 2016-11-17

**Authors:** Yinbing Pan, Xiaodi Sun, Lai Jiang, Liang Hu, Hong Kong, Yuan Han, Cheng Qian, Chao Song, Yanning Qian, Wentao Liu

**Affiliations:** 1 Department of Anesthesiology, The First Affiliated Hospital of Nanjing Medical University, Nanjing, Jiangsu 210029 People’s Republic of China; 2 Department of Pharmacology, Jiangsu Key Laboratory of Neurodegeneration, Nanjing Medical University, Nanjing, Jiangsu 210029 People’s Republic of China; 3 Jiangsu Province Key Laboratory of Anesthesiology, School of Anesthesiology, Xuzhou Medical College, Xuzhou, Jiangsu 221004 People’s Republic of China; 4 Department of Oncology, The Affiliated Hospital of Xuzhou Medical University, Xuzhou, Jiangsu 221000 People’s Republic of China

**Keywords:** Morphine tolerance, Glia activation, AMPK, Cytokines, MAPK

## Abstract

**Background:**

Tolerance seriously impedes the application of morphine in clinical medicine. Thus, it is necessary to investigate the exact mechanisms and efficient treatment. Microglial activation and neuroinflammation in the spinal cord are thought to play pivotal roles on the genesis and maintaining of morphine tolerance. Activation of adenosine monophosphate-activated kinase (AMPK) has been associated with the inhibition of inflammatory nociception. Metformin, a biguanide class of antidiabetic drugs and activator of AMPK, has a potential anti-inflammatory effect. The present study evaluated the effects and potential mechanisms of metformin in inhibiting microglial activation and alleviating the antinociceptive tolerance of morphine.

**Methods:**

The microglial cell line BV-2 cells and mouse brain-derived endothelial cell line bEnd3 cells were used. Cytokine expression was measured using quantitative polymerase chain reaction. Cell signaling was assayed by western blot and immunohistochemistry. The antinociception and morphine tolerance were assessed in CD-1 mice using tail-flick tests.

**Results:**

We found that morphine-activated BV-2 cells, including the upregulation of p38 mitogen-activated protein kinase (p38 MAPK) phosphorylation, pro-inflammatory cytokines, and Toll-like receptor-4 (TLR-4) mRNA expression, which was inhibited by metformin. Metformin suppressed morphine-induced BV-2 cells activation through increasing AMPK phosphorylation, which was reversed by the AMPK inhibitor compound C. Additionally, in BV-2 cells, morphine did not affect the cell viability and the mRNA expression of anti-inflammatory cytokines. In bEnd3 cells, morphine did not affect the mRNA expression of interleukin-1β (IL-1β), but increased IL-6 and tumor necrosis factor-α (TNF-α) mRNA expression; the effect was inhibited by metformin. Morphine also did not affect the mRNA expression of TLR-4 and chemokine ligand 2 (CCL2). Furthermore, systemic administration of metformin significantly blocked morphine-induced microglial activation in the spinal cord and then attenuated the development of chronic morphine tolerance in mice.

**Conclusions:**

Metformin significantly attenuated morphine antinociceptive tolerance by suppressing morphine-induced microglial activation through increasing AMPK phosphorylation.

**Electronic supplementary material:**

The online version of this article (doi:10.1186/s12974-016-0754-9) contains supplementary material, which is available to authorized users.

## Background

Morphine is the most commonly used drug for the treatment of severe pain. However, long-term morphine treatment leads to tolerance which greatly attenuates analgesic effect and diminishes clinical utilization. Therefore, investigating mechanisms of morphine tolerance and identification of solutions are of clinical significance.

Among the previous studies, mechanisms of morphine tolerance are complex and involve many factors, such as ion channels, receptors, cells, and neural networks [1–3]. For a long time, extensive studies suggested that neurons participate in the development of morphine tolerance [3, 4]. However, compelling evidences recently show that glia cells, especially microglia, play a pivotal role in the initiation and maintenance of morphine tolerance [5, 6].

Microglia in the spinal cord are significantly activated by chronic morphine treatment. Several studies showed that morphine induces a proinflammatory response through binding to Toll-like receptor 4 (TLR4), leading to initiation of the TLR4 signaling cascade, as do direct modulators of p38 and nuclear factor-κB (NF-κB), subsequently regulating the expression of multiple inflammation factors [7, 8]. Other studies established that activated microglia secrete large amounts of proinflammatory cytokines including interleukin-1β (IL-1β), IL-6, and tumor necrosis factor-α (TNF-α), which could enhance the hyperactivity of dorsal horn neurons, induce central sensitization, and reduce the antinociceptive effect of morphine [9, 10]. Thus, suppression of neuroinflammation by inhibiting microglial activation and proinflammatory cytokines could be a worthwhile strategy for enhancing morphine analgesic efficacy and attenuating morphine tolerance.

5′-Adenosine monophosphate-activated protein kinase (AMPK), a sensor of cellular energy change, regulates energy homeostasis and metabolic stress. Recent studies show that AMPK regulates both energy homeostasis and inflammatory defense [11–13]. Activation of AMPK inhibits ATP-consuming anabolic processes (such as protein translation) mainly via inhibiting mammalian target of rapamycin (mTOR) signaling [14]. AMPK activation also inhibits mitogen-activated protein kinase (MAPK) signaling. MAPK family, especially p38 mitogen-activated protein kinase (p38 MAPK) in activated microglia, have been shown to play an important role in morphine-induced neuroinflammation and tolerance [15]. In the brain, AMPK activation inhibits lipopolysaccharide (LPS)-induced pro-inflammatory cytokines expression by modulating NF-κB in primary rat microglia [16]. AMPK activation also inhibits the expression of pro-inflammatory mediators in the cerebral cortex of LPS-injected rats [11]. Thus, AMPK may be an interesting target for neuroprotective drugs in inflammatory conditions, such as morphine tolerance. We hypothesized that AMPK activation may represent a novel pharmacological treatment to reduce morphine tolerance by suppressing morphine-induced neuroinflammation through attenuating microglial activation.

To test our hypothesis, we used metformin, a potent antihyperglycaemic agent that has previously been shown to active AMPK, to assess the effect of AMPK activation on morphine-induced microglial activation and tolerance.

## Methods

### Animals

Adult CD-1 mice (18–22 g) were purchased from the Experimental Animal Center at Nanjing Medical University, Nanjing, China. Five to six mice per cage were housed under pathogen-free conditions with soft bedding under controlled temperature (22 ± 2 °C) and a 12-h light/dark cycle (lights on at 8:00 a.m.). For each group of experiments, the animals were matched by age and body weight. Behavioral testing was performed during the light cycle (between 9:00 a.m. and 5:00 p.m.). Mice were allowed to acclimate to these conditions for at least 2 days before inclusion in experiments.

### Reagents

All antibodies were purchased from Cell Signaling Technology (Beverly, MA, USA) unless stated otherwise. IL-1β was from Santa Cruz Biotechnology (Santa Cruz, CA, USA). Glyceraldehyde-3-phosphate dehydrogenase (GAPDH) and ionized calcium binding adapter molecule 1 (IBA-1) were from Sigma-Aldrich (St. Louis, MO, USA) and Wako Pure Chemical Industries (Osaka, Japan). The p65/RelA and immunofluorescence IBA-1 antibodies were from Abcam (Cambridge, MA, USA). Immunofluorescence c-fos and calcitonin gene-related peptide (CGRP) antibodies were from Cell Signaling Technology (Beverly, MA, USA) and Santa Cruz Biotechnology (Santa Cruz, CA, USA), respectively. Morphine hydrochloride was purchased from Shenyang First Pharmaceutical Factory, Northeast Pharmaceutical Group Company (Shenyang, China). Fetal bovine serum (FBS) and other cell culture media and supplements were purchased from Hyclone (Logan, UT, USA). 3-(4,5-Dimethyl-2-thiazolyl)-2,5-diphenyl-2-H-tetrazolium bromide (MTT) was purchased from Sunshine Biotechnology (Nanjing, China).

### Cell preparation and stimulation

BV-2 cells mouse brain endothelial cells bEND3 were cultured in humidified 5% CO_2_ at 37 °C in Dulbecco’s modified Eagle’s medium (DMEM) supplemented with 10% (*v*/*v*) FBS, penicillin (100 U/ml), and streptomycin (100 U/ml) (KeyGEN). For inducing inflammasome activation, 10^5^ cells were plated in 6-well plate overnight, and the medium were changed to serum-free medium next morning and then the cells were treated with morphine (200 μM) with or without metformin for 6 h. Metformin (4, 20, or 100 μM) was administrated 15 min before morphine treatment. Cell extracts and precipitated supernatants were analyzed by immunoblotting.

### Cell viability assessment

The cell viability was evaluated by CCK-8 assay (Dojindo Molecular Technologies, Inc.). BV-2 cells were plated in the 96-well plates (2.0 × 10^4^ cell per well) and incubated for 24 h before experiments. The cells were washed with D-Hanks buffer solution. Two hundred microliters of CCK-8 solution was added to each well and incubated for an additional 1 h at 37 °C. The optical density (OD) of each well at 450 nm was recorded on a Microplate Reader (Thermo, Varioskan Flash). The cell viability (% of control) is expressed as the percentage of (OD_test_ − OD_blank_) / (OD_control_ − OD_blank_), where OD_control_ is the optical density of the control sample and OD_blank_ is the optical density of the wells without BV-2 cells.

### Tolerance models and behavioral analysis

Animals was habituated in the testing environments for 2 days and carried out behavioral testing in a blinded manner. For the test of chronic tolerance, mice were injected with saline or morphine (10 mg/kg) subcutaneously every 12 h for 7 days and analgesia was assessed 30 min later by the tail-flick assay [17]. The test was performed by gently holding the mouse in a terry cloth towel and immersing between 2 and 3 cm from the tip of the tail into warm water (52 °C). A cutoff time of 10 s was set to avoid tissue damage. Data were calculated as percentage of maximal possible effect (% MPE), which was calculated by the following formula: 100% × [(Drug response time − Basal response time) / (10 s − Basal response time)] = % MPE. The experimenters were blinded to the treatment. Metformin (50, 100, or 200 mg/kg) was dissolved in saline and administered intraperitoneally 15 min before morphine treatment twice a day from day 1 to day 7.

### NF-κB activation assay

Cells (BV2) were plated in class bottom cell culture dishes and treated with morphine (200 μM) for 2 h with or without metformin (100 μM). Cells were fixed with ice-cold methanol and were permeabilized with 0.25% Triton X-100/PBST. After blocking with 1% bovine serum albumin (BSA) in PBST for 1 h, the coverslips with BV-2 cells were incubated for 2 h at room temperature with the p65/RelA antibody diluted in 1% BSA (1:50). Then, the coverslips were exposed to the fluorescein isothiocyanate (FITC)-conjugated antirabbit IgG (1:100, at room temperature for 1 h) and then were rinsed three times with PBS. Finally, the coverslips were stained with 1 μg/mL DAPI (4′,6-diamidino-2-phenylindole, a fluorescent DNA dye to mark nucleus) for 1 min. Confocal microscopy analyses were carried out using Olympus FV1000 confocal system.

### Analysis of mRNA levels by quantitative real-time polymerase chain reaction (PCR)

Cells samples were homogenized in Trizol reagent (Invitrogen Life Technologies, Carlsbad, CA, USA), and total RNA was treated by DNaseI and subjected to quantitative PCR, which was performed with ABI Prism 7300 sequence detection system (Applied Biosystems, Foster City, CA, USA) using SYBR Green I dye. The specific primer sequences for IL-1β, IL-6, TNF-α, TLR4, and GAPDH are listed as follows: IL-1β sense 5′-TCATTGTGGCTGTGGAGAAG-3′, antisense 5′-AGGCCACAGGTATTTTGTCG-3′, TNF-α sense 5′-CATCTTCTCAAAATTCGAGTGACAA-3′, antisense 5′-TGGGAGTAGACAAGGTACAACCC-3′, IL-6 sense 5′-ATCCAGTTGCCTTCTTGGGACTGA-3′, antisense 5′-TAAGCCTCCGACTTGTGAAGTGGT-3′, IL-4 sense 5′-CGAGGTCACAGGAGAAGG-3′, antisense 5′-TGAGGACGTTTGGCACAT-3′, TGF-β sense 5′-ATGGTGGACCGCAACAAC-3′, antisense 5′-GCACTGCTTCCCGAATGTC-3′, Toll-like receptor-4 (TLR-4) sense 5′-ACTGTTCTTCTCCTGCCTGACA-3′, antisense 5′-CCTAGTCTTTGAGTCGTTTCAGG-3′, IL-10 sense 5′-AACATACTGCTAACCGACTC-3′, antisense 5′-GGATCATTTCCGATAAGG-3′, GAPDH sense 5′-CAAAAGGGTCATCTCC-3′, and antisense 5′-CCCCAGCATCAAAGGTG-3′ GAPDH. Gene was used as an endogenous control to normalize for differences in the amount of total RNA in each sample.

### Western blot

To identify temporal expression level of IBA-1, GAPDH, IL-1β, TNF-α, and the phosphorylated protein levels of p38 MAPK, *N*-methyl-d-aspartic acid receptor NR1 (NMDAR-NR1), PKCγ, protein samples were analyzed as described before. In brief, samples (cells or spinal cord tissue segments at L1-L6) were collected and washed with ice-cold PBS before being lysed in radio immunoprecipitation assay (RIPA) lysis buffer [Beyotime, Shanghai, China; 50 mmol/L Tris (pH 7.4), 150 mmol/L NaCl, 1% Triton X-100, 1% sodium deoxycholate, 0.1% sodium dodecyl sulfate, 1 mmol/L phenylmethylsulfonyl fluoride, 0.15 U/mL aprotinin, and 1 mg/mL pepstatin] and then sample lysates were separated by SDS-PAGE and electrophoretically transferred onto polyvinylidene fluoride membranes (Millipore Corp., Bedford, MA, USA). The membranes were blocked with 10% whole milk in TBST (Tris-Hcl, NaCl, Tween 20) for 2 h at room temperature, probed with primary antibodies at 4 °C overnight [GAPDH, 1:8000; IBA-1, 1:1000; IL-1β, 1:1000; TNF-α, 1:1000; p-p38 (Tyr182), 1:1000; p-NR1(Ser896), 1:1000; p-PKCγ, 1:1000] and then incubated with horseradish peroxidase-coupled secondary antibodies from Cell Signaling Technology (Beverly, MA, USA). Data were acquired with the Molecular Imager (Gel DocTM XR, 170–8170) and analyzed with Quantity One-4.6.5 (Bio-Rad Laboratories, Berkeley, CA, USA).

### Immunofluorescence assay

After anesthesia by intraperitoneal injection of sodium pentobarbital (100 mg/kg), the animal was perfused with normal saline followed by 4% paraformaldehyde in 0.1 M PBS, pH 7.2–7.4, for 20 min. Then, L4 and/or L5 lumbar segment were dissected out and post-fixed in the same fixative. The embedded blocks were sectioned as 30 μm thick and processed for immunofluorescence assay. Sections from each group (five mice in each group) were incubated with primary antibody (IBA-1, 1:200; c-fos, 1:200; CGRP, 1:200). Then, the free-floating sections were washed with PBS and incubated with the secondary antibody (1:300; Jackson Laboratories, USA) for 2 h at room temperature. After using PBS to wash three times, the samples were investigated with a confocal microscope (Leica TCS SPEII, Leica Biosystems, Wetzlar, Germany) for morphologic details. Images were randomly coded and transferred to a computer for analysis.

### Statistical analysis

SPSS Rel 15 (SPSS Inc., Chicago, IL, USA) was used to conduct all the statistical analyses. Data were statistically evaluated by two-way analysis of variance (ANOVA) followed by Bonferroni post hoc tests. The mean fluorescent pixels of IBA-1 and CGRP were measured by Image Pro Plus 6.0 (Media Cybernetics, Silver Spring, MD, USA). Results were represented as mean ± SEM of three independent experiments. Results described as significant were based upon a criterion of *p* < 0.05.

## Results

### Metformin inhibits the inflammation induced by morphine in BV-2 cells

**Fig. 1:**
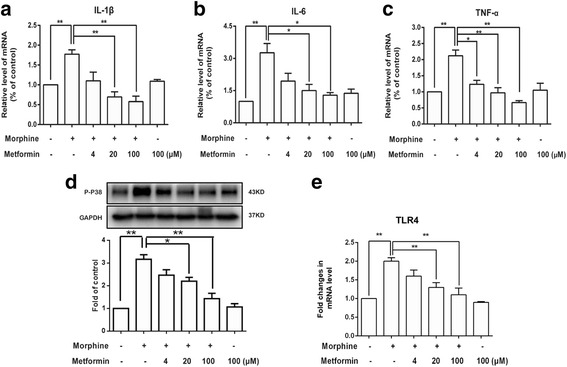
Metformin inhibited morphine-induced inflammation in microglial BV-2 cells. Cells were pretreated with metformin (4, 20, or 100 μM) for 15 min before the challenge of morphine (200 μM). BV-2 cells were collected and analyzed 6 h after morphine was given. **a**–**c** Metformin suppressed the expression of pro-inflammatory factors induced by morphine in BV-2 cells in a dose-dependent manner (*n* = 4). The levels of IL-1β, IL-6, and TNF-α mRNA were determined using real-time quantitative polymerase chain reaction (PCR). Glyceraldehyde 3-phosphate dehydrogenase (GAPDH) was used as an invariant control. **d** Metformin inhibited the phosphorylation of p38 induced by morphine treatment in BV-2 cells in a dose-dependent manner (*n* = 4). **e** Metformin suppressed TLR-4 mRNA expression induced by morphine in BV-2 cells in a dose-dependent manner (*n* = 4). The level of TLR-4 mRNA was determined with real-time quantitative PCR. (*p < 0.05; **p < 0.01 Bonferroni post hoc tests)

To investigate the effects of metformin on morphine-induced microglial activation in vitro, immortalized murine microglial cell line BV-2 cells were used. As shown in Fig. 1, morphine (200 μmol/L, 6 h) significantly induced the activation of BV-2 cells, which was characterized by up-regulated mRNA expression of pro-inflammatory cytokines (including IL-1β, IL-6, TNF-α, and TLR4), increased phosphorylation of p38 MAPK. Metformin administration before morphine significantly decreased these effects.

In addition, we also examined the potential effects of metformin on anti-inflammatory cytokines (including TGF-β, IL-4, and IL-10). Morphine did not affect the mRNA expression of anti-inflammatory cytokines, and metformin also had no significant effects on the expression of cytokines (Additional file 1 Fig. S1A, B and C). MTT test showed that morphine or metformin did not affect cell viability in BV-2 cells.

### Metformin inhibits microglial activation induced by morphine through AMPK activation

**Fig. 2:**
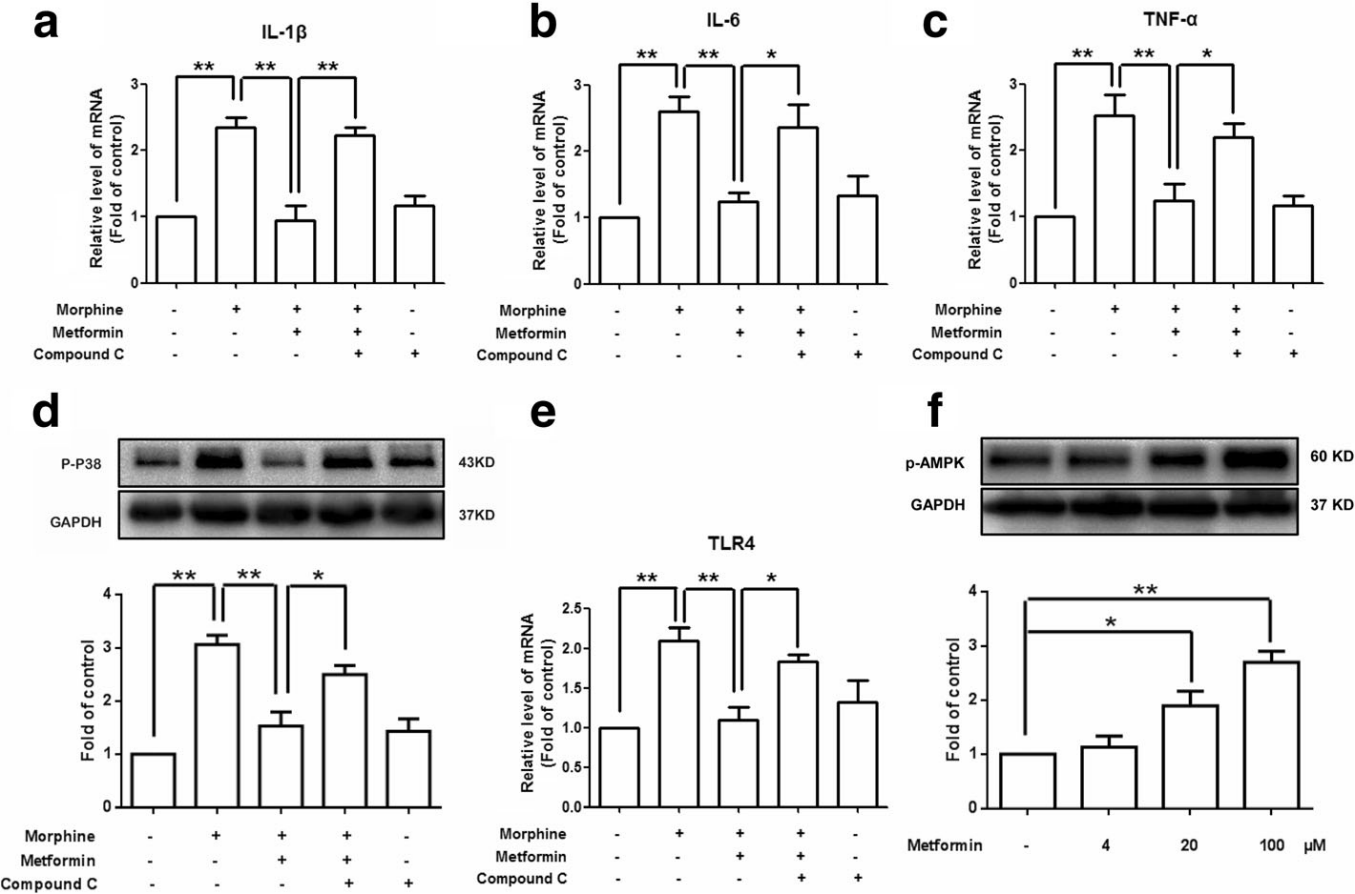
Metformin inhibits microglial activation induced by morphine through AMPK activation. **a**, **b**, **c**, **e** AMPK inhibitor compound C (20 μM) abolished the inhibition effects of metformin on the mRNA expression of IL-1β, IL-6, TNF-α, and TLR-4 in morphine-activated BV-2 cells (*n* = 4). **d** AMPK inhibitor compound C (20 μM) abolished the inhibition effects of metformin on the phosphorylation of p38 MAPK induced by morphine in BV-2 cells (*n* = 4). **f** Metformin increased phosphorylation of AMPK at Thr172 in BV2 cells in a dose-dependent manner. BV2 cells were collected and analyzed 6 h after incubation with metformin (4, 20, and 200 μM) (*n* = 4). (*p < 0.05; **p < 0.01; Bonferroni’s post hoc tests)

**Fig. 3:**
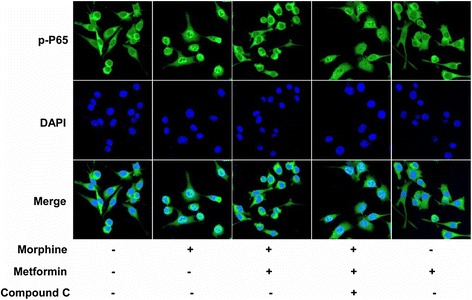
Metformin inhibited morphine-induced NF-κB (p65) activation in microglial BV-2 cells. Metformin (100 μM) attenuated the translocation of NF-κB (p65) from the cytosol to the nucleus after morphine treatment. The effects of metformin were abolished by the AMPK inhibitor compound C (*n* = 4). NF-κB activity was analyzed by p65 nuclear translocation assay. Original magnification, ×400. Significant difference was revealed following one-way analysis of variance (**p < 0.01; Bonferroni’s post hoc tests)

Previous studies have shown that metformin has been shown to function through activation of AMPK [18]. Therefore, we tested whether metformin inhibited microglial activation through AMPK activation in BV-2 cells. As shown in Fig. 2f, metformin increased phosphorylation of AMPK at Thr172 in a dose-dependent manner. Furthermore, we assessed whether AMPK inhibition could abolished the effects of metformin on morphine-induced microglia activation (Fig. 2a–e). We found that the AMPK inhibitor compound C significantly reversed the effects of metformin. Metformin inhibited the translocation of p65 NF-κB from the cytoplasm to the nucleus induced by morphine, which was also abolished by compound C (Fig. 3). These data showed that metformin inhibited microglial activation induced by morphine through AMPK signaling pathway.

### Metformin inhibits IL-6 mRNA expression in bEnd3 cells treated with morphine

**Fig. 4:**
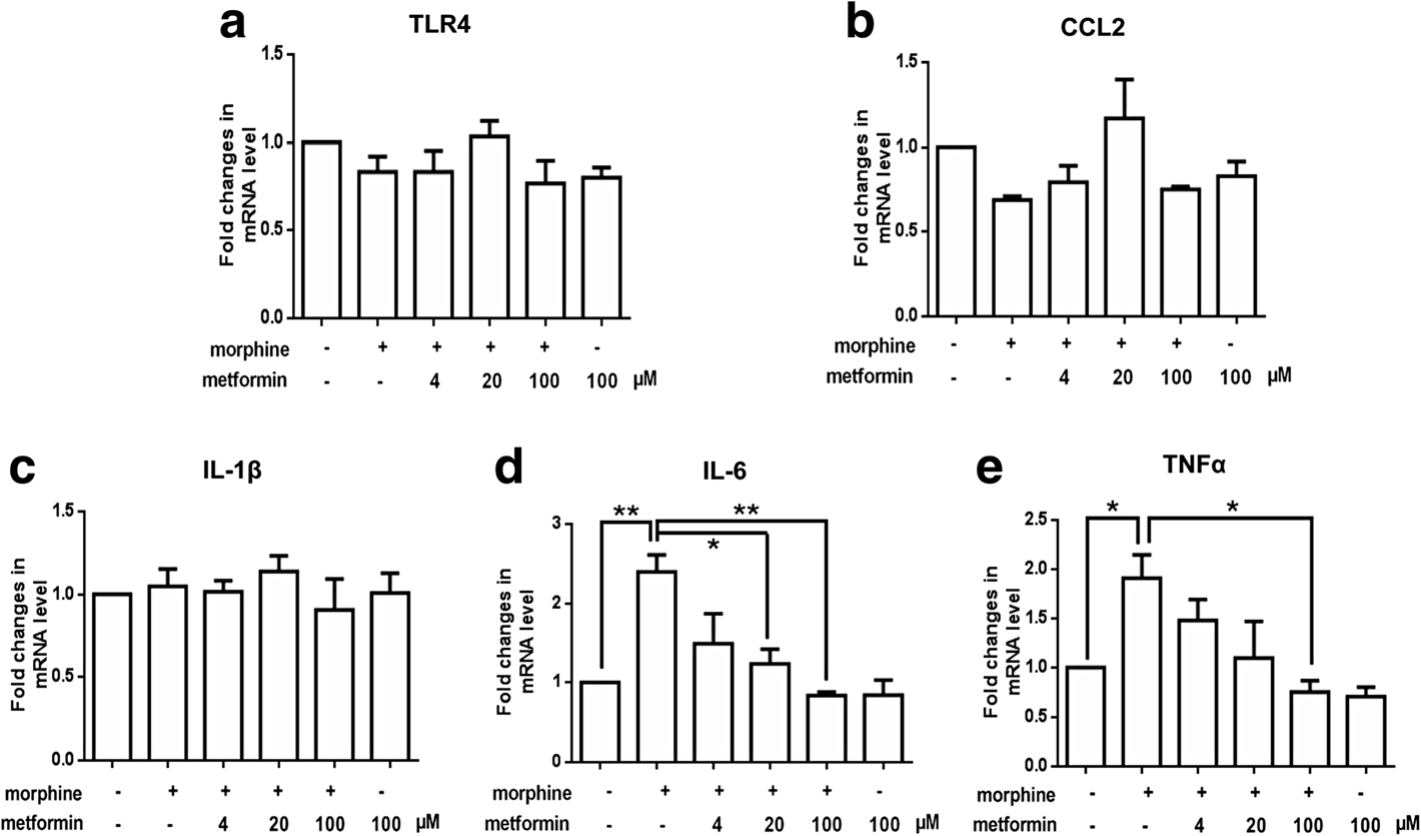
Metformin did not markedly affect morphine-induced inflammation in bEND3 cells. **a**–**c** Morphine with or without metformin treatment showed no notable effects on mRNA expression of TLR4, CCL2, and IL-1β in bEND3 cells (*n* = 4). **d**, **e** Morphine increased the mRNA expression of IL-6 and TNF-α, which were decreased by metformin treatment (100 and 200 μM) (*n* = 4). The levels of mRNA were determined using real-time quantitative polymerase chain reaction (PCR)

Recent studies have demonstrated that microvascular endothelial cells take part in the development of morphine-induced neuroinflammation and tolerance [19]. Therefore, we tested whether metformin regulate the expression of pro-inflammatory cytokines, chemokine ligand 2 (CCL2), and TLR4 in morphine-treated bEnd3 cells. As shown in Fig. 4, we found that morphine and metformin both did not affect the expression of TLR4, CCL2, and IL-1β, but metformin decreased the mRNA expression of IL-6 and TNF-α induced by morphine. These data showed that metformin did not markedly regulate microvascular endothelial cells when reducing morphine tolerance.

### Metformin attenuates chronic morphine tolerance in mice

**Fig. 5:**
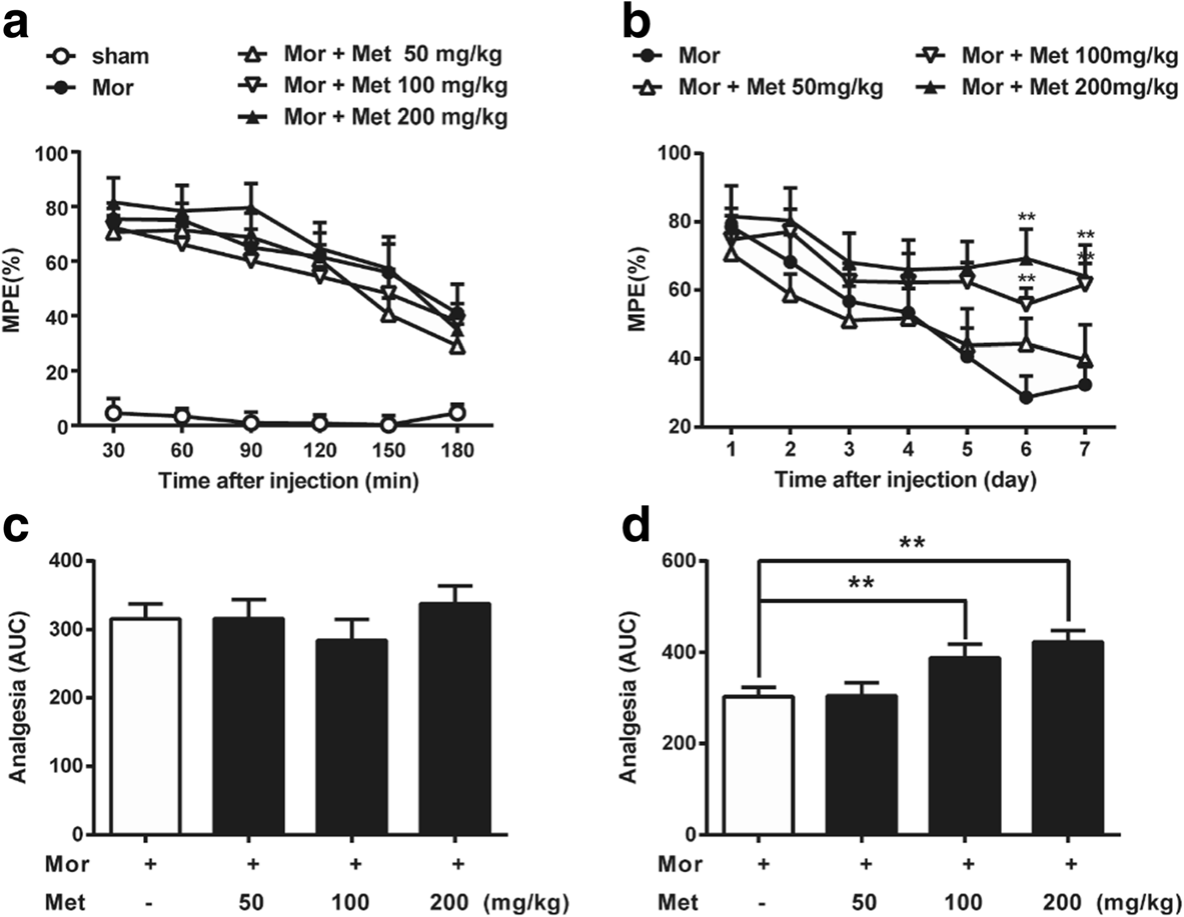
Metformin attenuated chronic morphine tolerance. Tail-flick method was performed to evaluate the effect of metformin on the morphine tolerance. Data were shown as percentage of maximal possible effect (% MPE). Twelve mice were included in each group. **a**, **c** Metformin (50, 100, and 200 mg/kg, i.p.) exhibited no effects on the initial analgesic response to morphine (10 mg/kg, s.c.) (*n* = 8). **b**, **d** Metformin suppressed chronic morphine tolerance (*n* = 8) (*p < 0.05; **p < 0.01; Bonferroni’s post hoc tests). Metformin was administered intraperitoneally daily 15 min before each morphine injection

To test the effects of metformin on morphine tolerance in vivo, i.t. injection of morphine (10 mg/kg, given subcutaneously each day for 7 days) was used to induce tolerance in mice. As shown in Fig. 5a, c, metformin [up to 200 mg/kg, intraperitoneal (i.p.), 15 min before each injection of morphine on each day] did not affect the pain threshold or the initial morphine-induced analgesia. However, repetitive metformin treatment significantly reduced the decreased analgesia resulted by morphine in a dose-dependent manner (Fig. 5b, d). These data showed that metformin is effective in attenuating chronic morphine tolerance.

### Metformin reduces spinal microglial activation in mice treated with chronic morphine

**Fig. 6:**
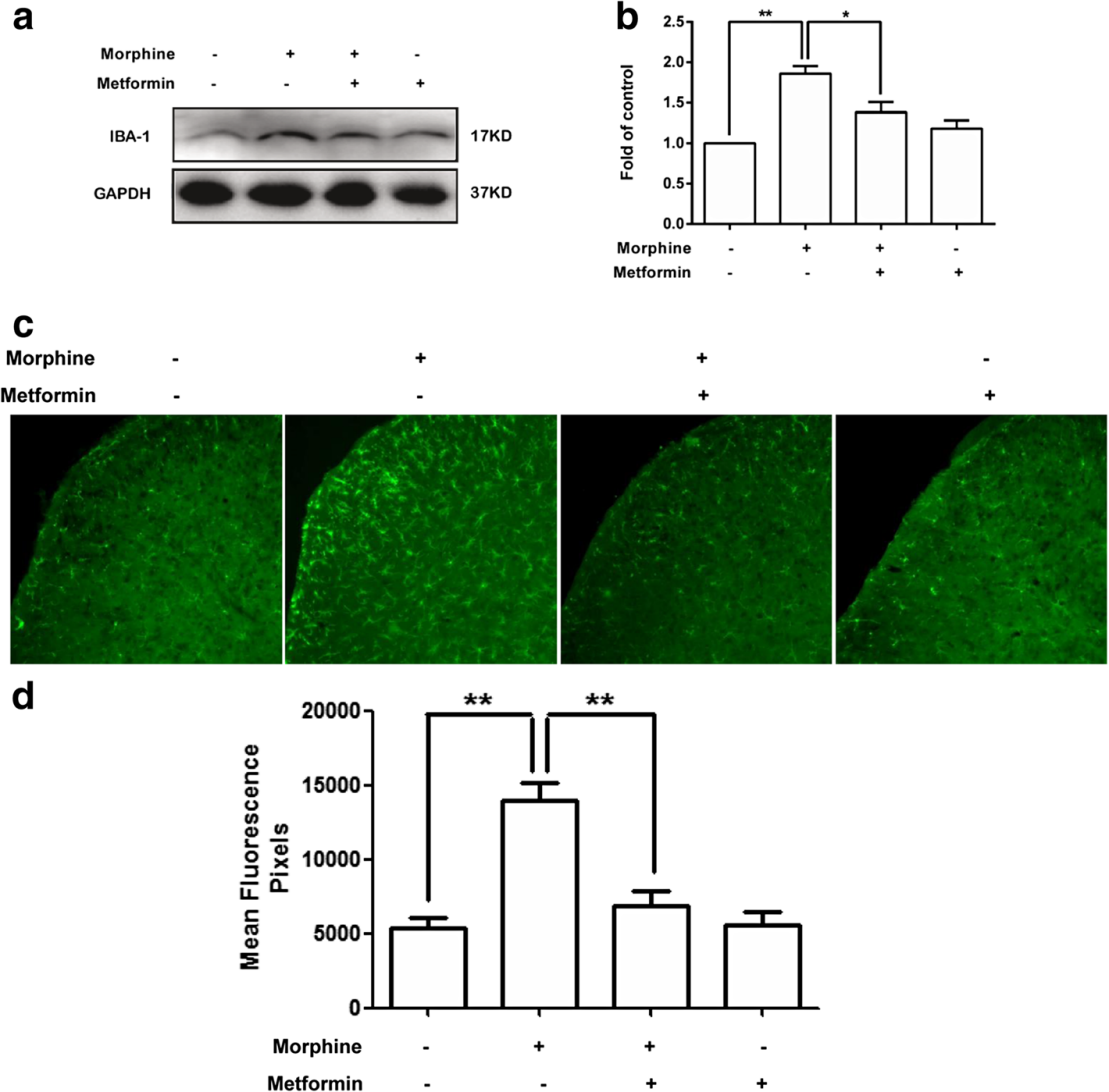
Metformin attenuated spinal microglial activation in chronic morphine administration. Morphine was injected subcutaneously (10 mg/kg) once a day for 7 days; metformin (200 mg/kg) was administered intraperitoneally daily 15 min before each morphine injection. The lumbar spines (L1–L6) were collected and analyzed 120 min after the last morphine administration. **a**, **b** Metformin inhibited up-regulation IBA-1 protein expression induced by chronic morphine treatment in the spinal cord (*n* = 4). **c**, **d** Metformin inhibited expression of IBA-1 induced by chronic morphine (*n* = 4). Confocal images and immunofluorescence analysis data showed IBA-1 in the dorsal horns (*scale bar*, 100 μm). Quantification of IBA1 immunofluorescence was represented as mean fluorescence pixels in the superficial dorsal horns (*n* = 5, 5 images per animal) (*p < 0.05; **p < 0.01; Bonferroni’s post hoc tests)

To investigate the mechanism of metformin in reducing morphine tolerance in vivo, we tested the expression of microglial activation marker IBA-1 in the spinal cord and measured the states of microglia. Our western blot and immunofluorescence data showed that repeated morphine exposure (10 mg/kg, given subcutaneously each day for 7 days) showed significant microglial activation in the spinal cord. The activation was manifested as an up-regulated IBA-1 protein level (Fig. 6a, b) and an increased IBA-1 fluorescence density (Fig. 6c, d). Metformin (200 mg/kg) significantly inhibited microglial activation induced by morphine. These data indicated that pretreatment with metformin could suppress the activation of spinal microglia induced by chronic morphine exposure in vivo.

**Fig. 7:**
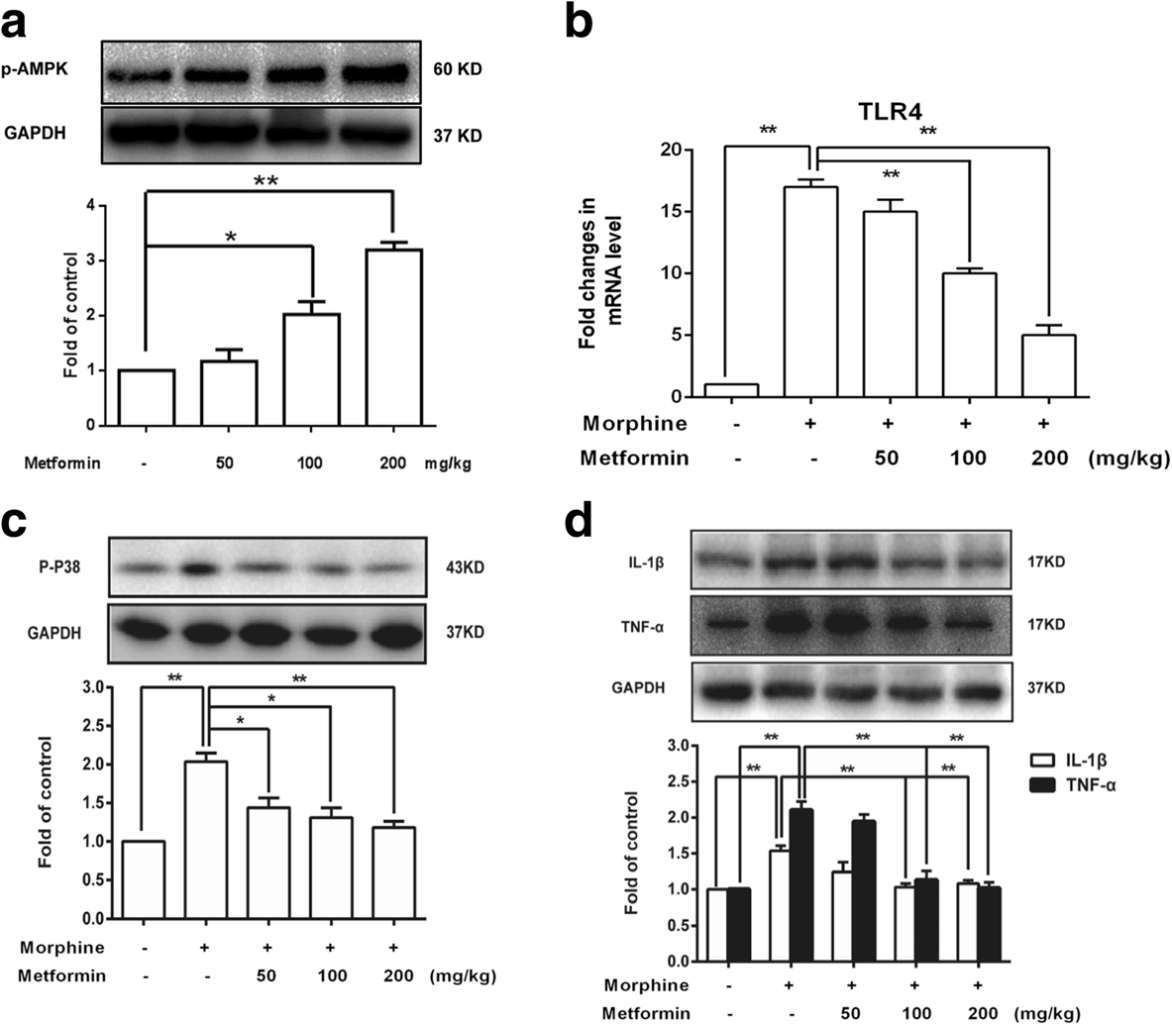
Metformin inhibits the inflammation induced by morphine in vivo. **a** Metformin increased AMPK-Tyr172 phosphorylation in mice spinal cords in a dose-dependent manner (*n* = 4). **b** Metformin inhibited morphine-induced up-regulation of TLR-4 mRNA in spinal cords (*n* = 4). **c** Metformin inhibited morphine-induced up-regulation of p38 phosphorylation in spinal cords (*n* = 4). **d** Metformin inhibited morphine-induced IL-1β and TNF-α protein expression in spinal cords (*n* = 4). The lumbar spines (L1–L6) were collected and analyzed 15 min after metformin (50, 100, and 200 mg/kg, i.p.) administration. Significant difference was revealed following one-way analysis of variance (*p < 0.05; **p < 0.01; Bonferroni’s post hoc tests)

Once activated, microglia release a large number of pro-inflammatory cytokines to induce inflammatory response. Therefore, we tested whether metformin regulate neuroinflammation induced by morphine. Similar to the results in vitro, metformin (50, 100, and 200 mg/kg) increased the phosphorylation of AMPK at Thr172 in a dose-dependent manner (Fig. 7a). Morphine significantly increased the expression of TLR4 mRNA, p-p38, IL-1β, and TNF-α protein in vivo, which were inhibited by metformin (100 and 200 mg/kg) (Fig. 7b–d). These data suggested that metformin could suppress neuroinflammation induced by morphine through AMPK-mediated signaling.

### Metformin attenutates central sensitization induced by morphine through reducing phosphorylation of NR1 and PKCγ

**Fig. 8:**
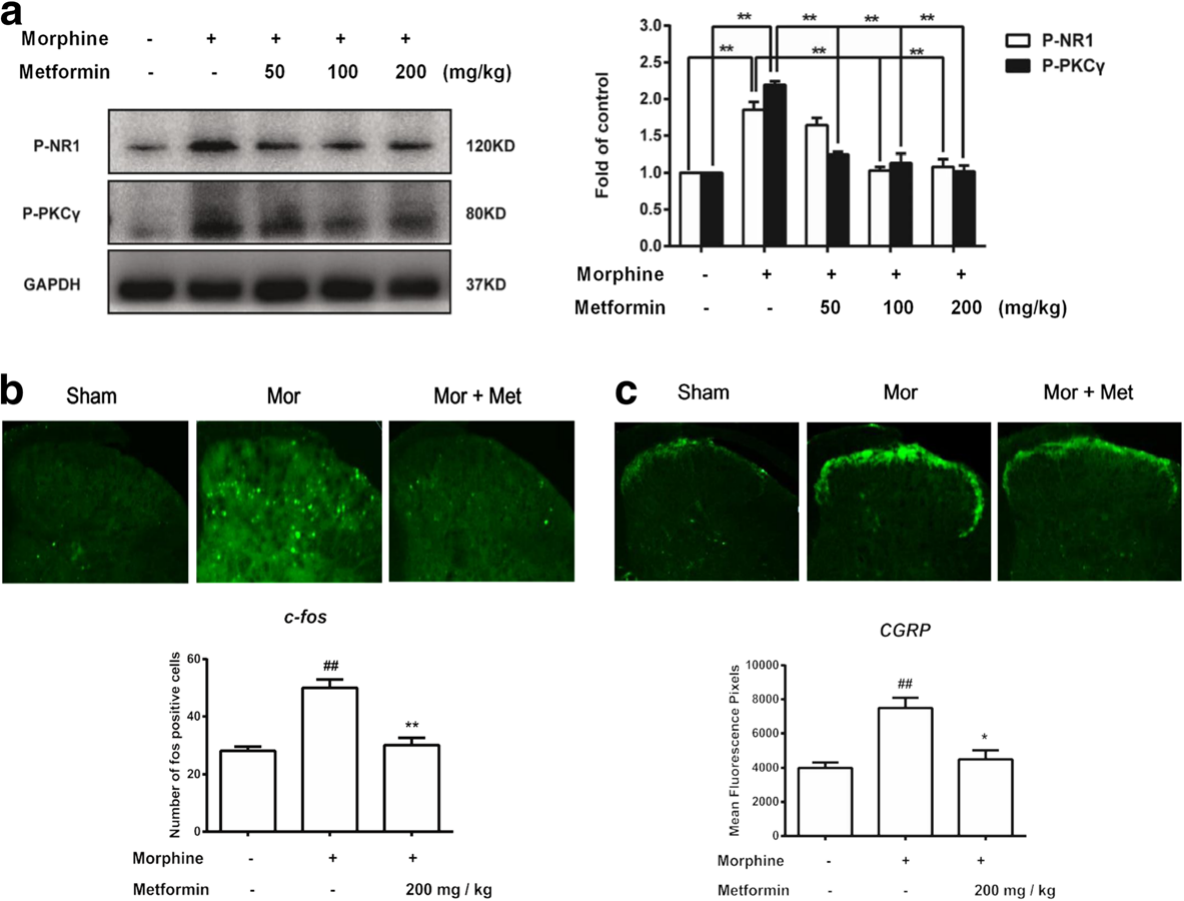
Metformin inhibits phosphorylation of c-fos and CGRP in the STN. **a** Metformin reduces morphine-induced phosphorylation of NR1 and PKCγ in vivo (*n* = 4). **b**, **c** Immunofluorescence analysis data show CGRP expression and c-fos-immunoreactive neurons number. The CGRP and c-fos level were significantly increased by morphine; metformin (200 mg/kg) suppressed the increase of CGRP expression and attenuated the increase of c-fos-immunoreactive neurons number. *n* = 5, five images per animal. Significant difference was revealed following one-way analysis of variance (*p < 0.05; **p < 0.01; Bonferroni’s post hoc tests)

The NMDA receptor, which regulates neuronal activity and synaptic efficacy, plays an important role in inflammatory response. NMDA receptor phosphorylation can be activated by pro-inflammatory factors and phosphorylated by protein kinase C (PKC). Thus, we examined whether metformin regulate phosphorylation of NR1 and PKCγ in the spinal cord to reduce inflammatory response. As shown in Fig. 8a, morphine markedly increased phosphorylation of NR1 and PKCγ, which were inhibited by metformin in a dose-dependent manner.

### Metformin inhibits phosphorylation of c-fos and CGRP in morphine-treated mice

Furthermore, we measured the expression of morphine tolerance indicators c-fos and CGRP in the spinal cord using immunofluorescence staining. As shown in Fig. 8b, the c-fos and CGRP level were significantly increased induced by morphine compared with sham group. Treatment with metformin (200 mg/kg) suppressed the increase of the c-fos and CGRP level. These results further explained that metformin could attenuate morphine tolerance in vivo.

## Discussion

In our study, we found that metformin, a potent antihyperglycaemic agent and AMPK activator, had a significant inhibitory effect on morphine-induced microglial activation. Metformin inhibited the morphine-induced up-regulation of p38 MAPK phosphorylation, NF-κB nuclear translocation, and proinflammatory cytokine expression in microglia, which were abolished by AMPK inhibition. Thus, metformin significantly attenuated the development of chronic morphine tolerance in an efficient manner. The study may provide a new solution by inhibiting microglial activation through increasing AMPK activation to improve clinical analgesic efficacy of morphine.

Non-neuronal cells, especially microglia, are crucial in the pathogenesis of morphine tolerance [20]. Microglial activation has been shown to play an important role in cytokine release in the CNS [21]. After chronic or acute exposure to morphine, activated microglia exhibit increased expression of pro-inflammatory cytokines, such as IL-1β, IL-6, TNF-α, and chemokines [22, 23]. These changes contribute to morphine analgesic tolerance. In the present study, we found that morphine increased the mRNA expression of IL-1β, IL-6, and TNF-α in BV-2 cells (Fig. 1a–c). Interestingly, metformin, a potent antihyperglycemic agent, was found to inhibit cytokines production induced by morphine (Fig. 1), and AMPK inhibition abolished the effects of metformin (Fig. 2a–c). These data suggest that metformin may be beneficial to reducing microglial activation and morphine tolerance.

Previous studies have shown that both pro-inflammatory and anti-inflammatory cytokines are involved in the development and maintenance of morphine tolerance [22, 24, 25]. IL-4, IL-10, and TGF-β are powerful anti-inflammatory cytokines with a wide spectrum of biological effects [26–28]. Therefore, we tested whether metformin inhibit inflammatory response via regulating anti-inflammatory cytokines production. In accordance with the previous study [26], we found that morphine did not affect the expression of anti-inflammatory cytokines mRNA level. However, metformin did not increase anti-inflammatory cytokines mRNA level (Fig. S1). These data suggest that the effects of metformin are mainly relates to the regulation of pro-inflammatory cytokines.

It is well known that NF-κB has diverse and complicated effects on the immune response and nervous systems [29, 30]. Activation of NF-κB is one of the major events following the onset of an inflammatory response mainly initiated by proinflammatory cytokines [31]. Activation of NF-κB also induces production of cytokines that activate NF-κB in cancer cells to induce chemokines that attract more inflammatory cells into the tumor [32]. Morphine can induce the translocation of NF-κB p65 from the cytosol to the nucleus, and NF-κB inhibition can reverse the mRNA expression of IL-1β, IL-6, and TNF-α following morphine treatment [33]. Therefore, NF-κB is an important transcription factor and plays critical roles in inflammation. Our study showed that morphine markedly increased NF-κB p65 level in the nucleus, which was reversed by metformin (Fig. 3). These data suggest that metformin may decrease the cytokines production via inhibiting activation of NF-κB.

Recent studies have demonstrated that the MAPK family, including p38 MAPK, extracellular signal-regulated protein kinase (ERK), and c-Jun N-terminal kinase, plays important roles in morphine tolerance [34–36]. The expression of pro-inflammatory cytokines and other harmful signaling molecules is regulated by p38 MAPK/NF-κB signaling pathway in the CNS [37]. Microglia inhibitor minocycline and p38 inhibitor SB203580 markedly attenuate morphine-induced pro-inflammatory cytokines production and inhibit morphine tolerance [38, 39]. We found that morphine increased p38 MAPK phosphorylation, which was decreased by metformin (Fig. 1d). The effect of metformin was abolished by AMPK inhibitor (Fig. 2d). In addition, compelling evidence has suggested that morphine induces microglial activation through binding with the Toll-like receptor 4 (TLR4) expressed in spinal microglia, activating downstream intracellular signaling pathways, leading to the release of cytokines and suppression of inflammatory response. TLR4 also plays a critical role in p38 phosphorylation induced by morphine. In our study, we found that morphine treatment significantly increased the mRNA expression of TLR4 in BV-2 cells, which was abolished by metformin (Fig. 1e). These findings suggest that TLR4/p38 MAPK signaling pathway was involved in the protection effects of metformin. Metformin inhibit microglial activation via reducing the morphine-induced mRNA expression of TLR 4 and proinflammatory cytokines. Consistent with our findings, Eidson and Murphy reported that blockade of TLR 4 attenuates morphine tolerance and facilitates the pain relieving properties of morphine [7].

Several studies have indicated that microvascular endothelial cells could be involved in morphine-induced neuroinflammation and play an important role [40, 41]. We tested whether metformin-attenuated morphine tolerance relate to regulating states of microvascular endothelial cells. We found that metformin did not affect the expression of CCL2, TLR4, and IL-1β. However, metformin significantly decreased the upregulation of IL-6 and TNF-α mRNA level induced by morphine (Fig. 4d, e). These data showed that regulation of IL-6 and TNF-α mRNA level by metformin in microvascular endothelial cells was partially involved in the attenuation of morphine tolerance.

Furthermore, we investigated whether metformin could attenuate morphine tolerance in vivo. In the present study, behavioral tests showed that the mice developed allodynia and hyperalgesia following morphine withdrawal (Fig. 5). Metformin reduced chronic morphine tolerance in a dose-dependent manner. Previous studies have shown that expression of the microglial maker IBA-1 is significantly increased when microglia are activated. Our data showed that resveratrol notably inhibited microglial activation, suppressing the up-regulated IBA-1 expression in the spinal dorsal horn (Fig. 6).

The NMDA receptor, regulating neuronal activity and synaptic efficacy, plays an important role in various inflammation states [21]. The NMDA receptor 1 (NR1) is preferentially phosphorylated by PKCγ [42]. PKCγ activation plays well-developed role on central sensitization in morphine tolerance, which may contribute to increasing the excitability of nociceptive neurons [43]. Our results showed that metformin provide an inhibition in the activation of PKCγ and the phosphorylation of NMDA receptors NR1 in morphine tolerance mice (Fig. 8a). These data suggest that metformin may inhibit microglial activation and further suppress central sensitization occurring in the spinal cord, which contribute to the attenuation of morphine tolerance. In addition, calcitonin gene-related peptide (CGRP) and c-fos have been implicated in pain transmission and morphine tolerance [44–46] and have been considered as the indicators of morphine tolerance. It is widely distributed in central nervous system and peripheral organs in rodents. Our group has demonstrated that the increase of CGRP and c-fos release induced by morphine could be almost completely abolished by metformin (Fig. 8b). These data suggest that metformin could be an operative medicine to attenuate morphine tolerance.

## Conclusions

In this study, we provided the evidence for the first time that metformin, a biguanide class of antidiabetic drugs, could extend morphine analgesia and improve chronic morphine tolerance. We have illustrated the possible mechanism by which metformin inhibited microglial activation and attenuated morphine tolerance. Our data showed that metformin inhibits microglial activation and relieves microglia-mediated neuroinflammation via AMPK activation and further suppresses central sensitization occuring in the spinal cord and reduces morphine tolerance. Therefore, our study may potentially offer a new clinical application of metformin in morphine tolerance and suggest that AMPK may be a target of clinical treatment in attenuating morphine tolerance.

## Additional files

Additional file 1: Metformin reduces morphine tolerance by inhibiting microglial-mediated neuroinflammation. (DOCX 198 kb)

## Abbreviations

AMPK: Adenosine monophosphate-activated kinase
BSA: Bovine serum albumin
CCL2: Chemokine ligand 2
CGRP: Calcitonin gene-related peptide
DMEM: Dulbecco’s modified Eagle’s medium
FBS: Fetal bovine serum
GAPDH: Glyceraldehyde-3-phosphate dehydrogenase
IBA-1: Ionized calcium binding adapter molecule 1
IL-1β: Interleukin-1β
LPS: Lipopolysaccharide
MAPK: Mitogen-activated protein kinase
mTOR: Mammalian target of rapamycin
MTT: 3-(4,5-Dimethyl -2-thiazolyl)-2,5-diphenyl -2-H-tetrazolium bromide
NF-κB: Nuclear factor-κB
NMDAR-NR1: *N*-Methyl-Daspartic acid receptor NR1
p38 MAPK: p38 mitogen-activated protein kinase
TLR-4: Toll-like receptor-4
TNF-α: Tumor necrosis factor-α
