# Supplementary material for: Metformin reduces morphine tolerance by inhibiting microglial-mediated neuroinflammation
Source: J Neuroinflammation. 2016 Nov 17;13:294. doi: 10.1186/s12974-016-0754-9 (PMC5114746; doi:10.1186/s12974-016-0754-9)
Supplement: Additional file 1: — Metformin reduces morphine tolerance by inhibiting microglial-mediated neuroinflammation. (DOCX 198 kb) [file 12974_2016_754_MOESM1_ESM.docx]

**Metformin reduces morphine tolerance by inhibiting microglial-mediated neuroinflammation**

Yinbing Pan^1*^, Xiaodi Sun^1*^, Lai Jiang^2^, Liang Hu^2^, Hong Kong^2^, Yuan Han^3^, Cheng Qian^2^, Chao Song^4^, Yanning Qian^1^, Wentao Liu^2#^

^1^Department of Anesthesiology, The First Affiliated Hospital of Nanjing Medical University, Nanjing, Jiangsu 210029, People’s Republic of China.

^2^Jiangsu Key Laboratory of Neurodegeneration, Department of Pharmacology, Nanjing Medical University, Nanjing, Jiangsu 210029, People’s Republic of China.

^3^Jiangsu Province Key Laboratory of Anesthesiology, School of Anesthesiology, Xuzhou Medical College, Xuzhou, Jiangsu 221004, People’s Republic of China.

^4^Department of Oncology, The Affiliated Hospital of Xuzhou Medical University, Xuzhou, Jiangsu 221000 People’s Republic of China

^*^Equal contributors

^#^Corresponding author: painresearch@njmu.edu.cn.

**Figure S1**


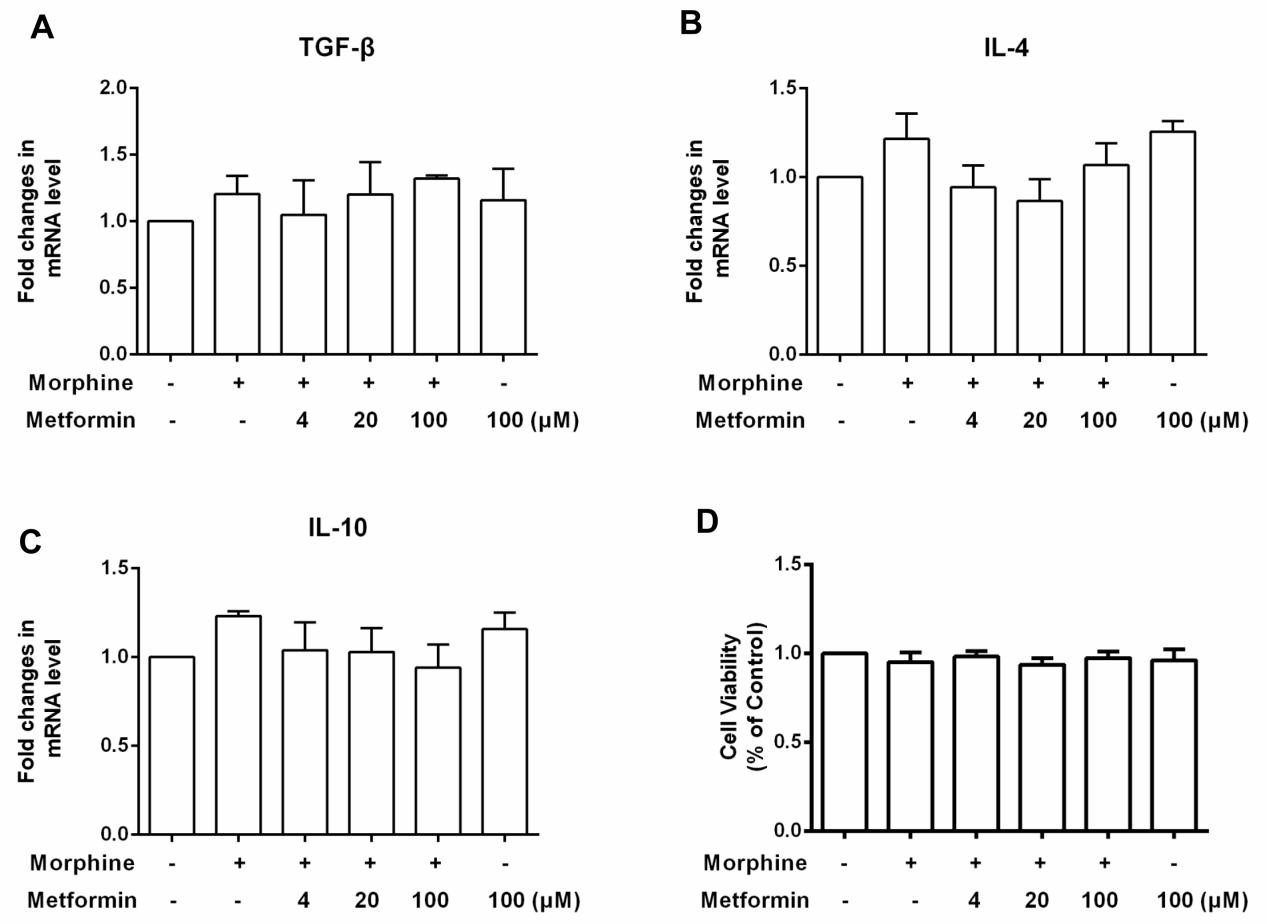


**Figure S1 Metformin did not affect morphine-induced anti-inflammatory cytokines in BV-2 cells and cell viability.** (A) (B) (C) Morphine with or without metformin treatment showed no notable effects on mRNA expression of TGF-β, IL-4 and IL-10 in BV-2 cells (n = 4). (D) Morphine with or without metformin treatment did not affect cell viability (n=4).

**Figure S2**


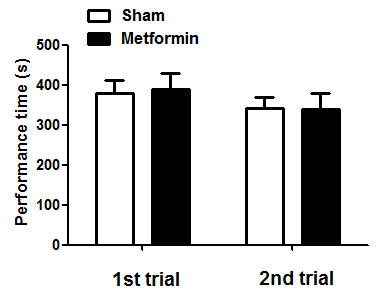


**Figure S2 Metformin treatment did not affect motor performance in mice.** Rotarod tests were performed 30 (first trial) and 60 min (second trial) after metformin (200mg/kg) administration. There was no significant change in the average latency of time to fall from the rotarod in each group. Data represent mean ± SEM (n=5-6). *p < 0.05, compared to vehicle sham (Sham).
